# The critical amplifying role of increasing atmospheric moisture demand on tree mortality and associated regional die-off

**DOI:** 10.3389/fpls.2013.00266

**Published:** 2013-08-02

**Authors:** David D. Breshears, Henry D. Adams, Derek Eamus, Nate G. McDowell, Darin J. Law, Rodney E. Will, A. Park Williams, Chris B. Zou

**Affiliations:** ^1^The School of Natural Resources and the Environment, The University of ArizonaTucson, AZ, USA; ^2^Department of Ecology and Evolutionary Biology, The University of ArizonaTucson, AZ, USA; ^3^Earth and Environmental Sciences Division, Los Alamos National LaboratoryLos Alamos, NM, USA; ^4^School of the Environment, University of Technology SydneySydney, NSW, Australia; ^5^Department of Natural Resource Ecology and Management, Oklahoma State UniversityStillwater, OK, USA

Drought-induced tree mortality, including large-scale die-off events and increases in background rates of mortality, is a global phenomenon (Allen et al., 2010) that can directly impact numerous earth system properties and ecosystem goods and services (Adams et al., 2010; Breshears et al., 2011; Anderegg et al., 2013). Tree mortality is particularly of concern because of the likelihood that it will increase in frequency and extent with climate change (McDowell et al., 2008, 2011; Adams et al., 2009; McDowell, 2011; Williams et al., 2013). Recent plant science advances related to drought have focused on understanding the physiological mechanisms that not only affect plant growth and associated carbon metabolism, but also the more challenging issue of predicting plant mortality thresholds (McDowell et al., 2013). Although some advances related to mechanisms of mortality have been made and have increased emphasis on interrelationships between carbon metabolism and plant hydraulics (McDowell et al., 2011), notably few studies have specifically evaluated effects of increasing atmospheric demand for moisture (i.e., vapour pressure deficit; VPD) on rates of tree death. In this opinion article we highlight the importance of considering the key risks of future large-scale tree die-off and other mortality events arising from increased VPD. Here we focus on mortality of trees, but our point about the importance of VPD is also relevant to other vascular plants.

Much research discussion has stemmed from speculation that warmer temperatures and implicit increases in VPD exacerbated a recent widespread dieoff event of one tree species, *Pinus edulis*, in the southwestern USA (Breshears et al., 2005). This speculation was subsequently supported by theoretical developments regarding related processes (McDowell et al., 2008; McDowell, 2011), a controlled experiment (Adams et al., 2009), and regional empirical and modeling analyses (Weiss et al., 2012; Jiang et al., 2013; Williams et al., 2013). Numerous other studies reached similar conclusions for other systems (e.g., Allison et al., 2009; van Mantgem et al., 2009; Arora et al., 2013; Jiang et al., 2013; Liu et al., 2013). From a variety of approaches, these studies collectively concluded that rising temperature and associated VPD drive accelerating rates of mortality during drought. Although the effects of warmer temperature are receiving increased attention, effects of changes in VPD *per se* have been explicitly considered far less.

To focus on VPD, we return to fundamental relationships. Rising global surface temperature will curvilinearly increase saturation vapour pressure because warming increases the evaporation-to-condensation ratio, causing the gaseous phase of water to be increasingly favored (Bohren and Albrecht, 1998). VPD is the difference between the saturation vapour pressure and actual vapour pressure. While increased temperature is often accompanied by increased vapour pressure due to enhanced evaporation rates, increases in saturation vapour pressure outpace increases in actual vapour pressure as long as relative humidity is less than 100%, resulting in a curvilinear increase in VPD (Figure 1A). Consequently as global climate warms, VPD increases even though specific humidity is projected to increase in most regions (Held and Soden, 2006). Drought induces an additional positive feedback on VPD due to an increase in the ratio of sensible to latent heat fluxes caused by reduced transpiration and evaporation, thus causing a further rise in surface temperature and hence VPD (Maness et al., 2013).

**Figure 1:**
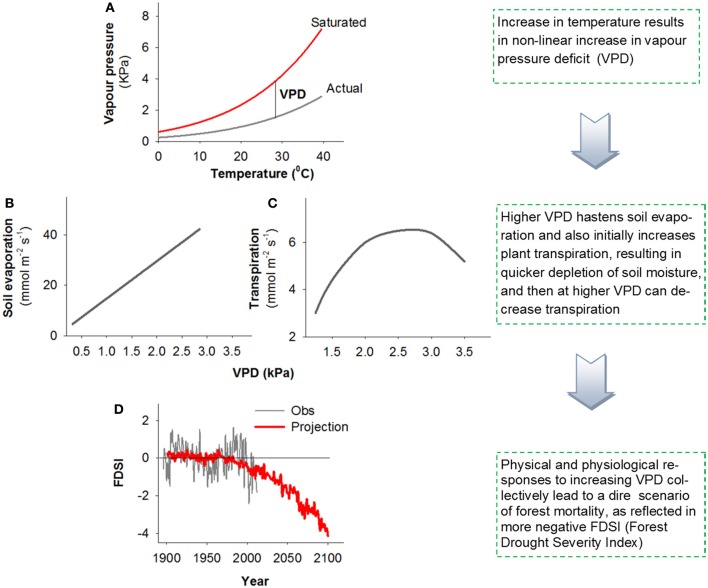
**A conceptual figure illustrating the effect of increased VPD on the biophysical factors that influence tree physiology, drought stress, and survival.** Higher temperatures increase VPD non-linearly **(A)**, higher VPD will generally both deplete soil moisture **(B)** and increase plant stress though changes in transpiration [**C**; based on data from Eamus et al. (2008)], all of which are projected to contribute to non-linear increases in forest stress [highlighted by the Forest Drought Severity Index (FDSI), with more negative values corresponding to increased stress] and resultant widespread regional mortality (**D**; Williams et al., 2013).

Climate-model projections of rapidly increasing VPD globally may cause pronounced levels of tree stress that may be unprecedented relative to what modern forests have evolved under, and for which landscape management strategies have been designed (e.g., Williams et al., 2013). If such stresses trigger associated widespread mortality, they then also have major impacts on landscape albedo and hence energy partitioning, biogeochemical cycling, regional carbon and water budgets, and the provisioning of ecosystem services (Adams et al., 2010; Breshears et al., 2011; Anderegg et al., 2013). Importantly, warmer temperatures and associated increases in VPD are two of the most pronounced climate change trends of recent decades and may be the climatic parameters that we can project with greatest confidence (IPCC, 2007). Therefore the need to understand the effects of increased VPD and temperature on forest stress and associated mortality is clearly apparent.

An increase in VPD affects both soil evaporation and plant physiology. Soil evaporation is affected by physical relationships with increased VPD under warmer temperature via Fick's law: E = *g* (VPD), where *g* is conductance of the surface boundary layer (Figure 1B). This causes increased rates of potential water loss from soils, thus reducing the amount of plant available water, which in turn could exacerbate plant water stress and associated mortality risk. In addition to this effect on soil evaporation, VPD affects plant physiology directly through its impact on stomatal closure and associated impacts on photosynthesis and carbon metabolism [Figure 1C; example data from Eamus et al. (2008)], transpiration rate increases with VPD up to a point (after which it remains high even if decreasing somewhat with VPD), the net result of which is likely a further exacerbation of plant water stress. As VPD rises, a series of physiological mechanisms may occur by which stomata close to maintain water tension within the xylem below a critical threshold (Tardieu and Simonneau, 1998). Such stomatal closure causes a reduction or cessation of photosynthesis, but failure to close stomata may cause desiccation through excessive water loss. These relationships were the basis for the carbon starvation and hydraulic failure hypotheses (McDowell et al., 2008, 2011): a reduction in photosynthesis, if prolonged and severe, should cause a decline in photosynthate available to drive metabolism and defense against biotic agents. Alternatively, if stomata remain relatively open during periods of elevated VPD, this may allow maintenance of positive photosynthetic rates, but may allow transpiration to exceed critical rates causing xylem cavitation—the formation of embolized vessels through the entry and expansion of air bubbles that block water transport (Tyree and Sperry, 1989; Thomas and Eamus, 1999). If embolized conduits remain un-repaired, this can lead to hydraulic failure, or dehydration and subsequent mortality (McDowell et al., 2008). Carbon starvation and hydraulic failure are likely interactive in driving mortality through multiple pathways (see Box 2, Figure 1 in McDowell et al., 2011), as supported by recent work for several species (Anderegg et al., 2012; Adams et al., 2013; Galvez et al., 2013; Quirk et al., 2013; Sevanto et al., 2013). Increased VPD can both increase xylem tensions that lead to hydraulic failure and inhibit phloem function, limiting the mobility of carbohydrate resources to sink tissues, potentially exacerbating carbon starvation and preventing xylem embolism repair (McDowell et al., 2011).

Note that the VPD effects on physiology described above do not negate the direct negative impact rising temperature alone can have on plant survival. Although growth respiration is reduced during drought (Amthor and McCree, 1990; Körner, 2003; McDowell, 2011), the increased temperatures associated with higher VPD may increase maintenance respiration, potentially accelerating carbon starvation (Adams et al., 2009). Further, rising temperature may also increase the speed to reproductive maturation of biotic agents such as bark beetles, thus increasing the rate of biotic attack on vegetation (Raffa et al., 2008). Recent modeling analysis disaggregated the effects of VPD and temperature on tree physiology in *Eucalyptus* (Eamus et al., 2013) and demonstrated that increased VPD (+1.0 or +2.5 kPa above controls) should have a much larger impact on tree health (defined as a prolonged loss of NPP, Net Primary Productivity) than increased temperature (+2.0 or +5.0 °C above controls). Similarly, in an experimental study, increased VPD associated with higher temperature led to greater transpiration and faster mortality during drought for tree seedlings common to the Great Plains forest-grassland ecotone of the central United States (Will et al., 2013). These modeling and experimental results are consistent with recent studies relating spatial patterns in VPD anomalies to tree die-off (Weiss et al., 2009, 2012). Additionally, powerful new relationships detected among regional tree growth, mortality and warm-season VPD portend non-linear increases in forest stress and associated tree mortality in the future (Figure 1D, as reflected in the Forest Drought Severity Index, FDSI, that includes a term for atmospheric demand; Williams et al., 2013) Collectively the physical (Figures 1A,B) and physiological (Figure 1C) effects of VPD are expected to contribute to greater water loss rates from the system and associated increases in tree drought stress and associated mortality.

The fundamental fine-scale relationships of increased temperature and associated increased VPD have profound global-scale implications for the future distributions of vegetation. VPD and temperature are core constituents of the three principle climatic determinants of the distributional patterns of vegetation, as exemplified in the Holdridge (1967) life-zone classification scheme: temperature, rainfall and potential evapotranspiration (the latter of which is strongly dependent on VPD). The potential for major redistribution of ecosystem boundaries following changes in evaporative demand without concomitant changes in rainfall is exemplified by the southern boundaries of boreal forest and aspen parkland in Canada, which correspond most closely with climatic (rainfall and atmospheric water content) moisture regimes (annual precipitation minus potential evaporation; Hogg, 1994). In general, there is a paucity of mortality experiments that manipulate either temperature or VPD, let alone both independently, and the former of these have mostly been limited to *Pinus* and *Eucalyptus*. Needed to compliment more mechanistic approaches are also experimentally determined climate-mortality envelopes that are specific to drought-induced tree mortality. Although much uncertainty remains about the specifics of the mechanisms underlying mortality, our principle point is that the risk posed by intensifying atmospheric moisture demands to future tree mortality and associated die-off events remains a critical but little-studied aspect in this domain. Additional study is required if we are to effectively predict and manage the consequences of future climate change and tree mortality. In summary, we need to shift focus to the critical amplifying role of VPD, not just of associated temperature, in driving tree mortality during drought because VPD changes impose fundamental curvilinear physical and physiological responses. Importantly climate models consistently predict VPD as well as temperature to increase in the future and these trends will almost certainly increase forest stress, tree mortality, and associated large-scale tree die-off events in many regions globally.

## References

[B3] Adams H. D. Germino M. J. Breshears D. D. Barron-Gafford G. A. Guardiola-Claramonte M. Zou C. B. (2013). Nonstructural leaf carbohydrate dynamics of *Pinus edulis* during drought-induced tree mortality reveal role for carbon metabolism in mortality mechanism. New Phytol. 197, 1142–1151 10.1111/nph.12102 23311898

[B1] Adams H. D. Guardiola-Claramonte M. Barron-Gafford G. A. Villegas J. C. Breshears D. D. Zou C. B. (2009). Temperature sensitivity of drought-induced tree mortality portends increased regional die-off under global-change-type drought. Proc. Natl. Acad. Sci. U.S.A. 106, 7063–7066 10.1073/pnas.0901438106 19365070 PMC2678423

[B2] Adams H. D. Macalady A. K. Breshears D. D. Allen C. D. Stephenson N. L. Saleska S. R. (2010). Climate-induced tree mortality: earth system consequences. Eos 91, 153–154 10.1029/2010EO170003

[B4] Allen C. D. Macalady A. K. Chenchouni H. Bachelet D. McDowell N. Vennetier M. (2010). A global overview of drought and heat-induced tree mortality reveals emerging climate change risks for forests. For. Ecol. Manag. 259, 660–684 10.1016/j.foreco.2009.09.001

[B5] Allison I. Bindoff N. L. Bindschadler R. A. Cox P. M. de Noblet N. England M. H. (2009). The Copenhagen Diagnosis 2009: Updating the World on the Latest Climate Science. Oxford: Elsevier

[B6] Amthor J. S. McCree K. J. (1990). Carbon balance of stressed plants: a conceptual model for integrating research results, in Stress Responses in Plants: Adaptation and Acclimation Mechanisms, eds Alscher R. G. Cumming J. R. (New York, NY: Wiley-Liss), 1–15

[B7] Anderegg W. R. L. Berry J. A. Smith D. D. Sperry J. S. Anderegg L. D. L. Field C. B. (2012). The roles of hydraulic carbon stress in a widespread climate-induced forest die-off. Proc. Natl. Acad. Sci. U.S.A. 109, 233–237 22167807 10.1073/pnas.1107891109 PMC3252909

[B8] Anderegg W. R. L. Kane J. Anderegg L. D. L. (2013). Consequences of widespread tree mortality triggered by drought and temperature stress. Nat. Clim. Change 3, 30–36 10.1038/nclimate1635

[B9] Arora V. K. Boer G. J. Friedlingstein P. Eby M. Jones C. D. Christian J. R. (2013). Carbon-concentration and carbon-climate feedbacks in CMIP5 Earth system models. J. Clim. (in press). 10.1175/JCLI-D-12-00494.1

[B10] Bohren C. F. Albrecht B. A. (1998). Atmospheric Thermodynamics. New York, NY: Oxford University Press

[B11] Breshears D. D. Cobb N. S. Rich P. M. Price K. P. Allen C. D. Balice R. G. (2005). Regional vegetation die-off in response to global-change type drought. Proc. Natl. Acad. Sci. U.S.A. 102, 15144–15148 10.1073/pnas.0505734102 16217022 PMC1250231

[B12] Breshears D. D. López-Hoffman L. Graumlich L. J. (2011). When ecosystem services crash: preparing for big, fast, patchy climate change. Ambio 40, 256–263 10.1007/s13280-010-0106-4 21644454 PMC3357807

[B13] Eamus D. Boulain N. Cleverly J. Breshears D. D. (2013). Global change-type drought-induced tree mortality: vapour pressure deficit is more important than temperature *per se* in causing decline of tree health. Ecol. Evol. [Epub ahead of print]. 10.1002/ece3.664 PMC3930053 24567834

[B14] Eamus D. Taylor D. T. MacInnis-NG C. M. O. Shanahan S. De Silva L. (2008). Comparing model predictions and experimental data for the response of stomatal conductance and guard cell turgor to manipulations of cuticular conductance, leaf-to-air vapour pressure difference and temperature: feedback mechanisms are able to account for all observations. Plant Cell Environ. 31, 269–277 10.1111/j.1365-3040.2007.01771.x 18088329

[B15] Galvez D. A. Landäusser S. M. Tyree M. T. (2013). Low root reserve accumulation during drought may lead to winter mortality in poplar seedlings. New Phytol. 198, 139–148 23347066 10.1111/nph.12129

[B16] Held I. M. Soden B. J. (2006). Robust responses of the hydrological cycle to global warming. J. Clim. 19, 5686–5699 10.1175/JCLI3990.1

[B17] Hogg E. H. (1994). Climate and the southern limit of the western Canadian boreal forest. Can. J. For. Res. 24, 1835–1845 10.1139/x94-237

[B18] Holdridge L. R. (1967). Life zone ecology. San Jose, CA: Tropical Science Center

[B19] IPCC. (2007). Climate Change 2007: Synthesis Report, in Contribution of Working Groups I, II and III to the Fourth Assessment Report of the Intergovernmental Panel on Climate Change, eds Core Writing Team, Pachauri R. K. Reisinger A. (Geneva: IPCC), 104

[B20] Jiang X. Rauscher S. Ringler T. Lawrence D. Williams A. Allen C. (2013). Projected future changes in vegetation in western North America in the 21st century. J. Clim. 26, 3671–3687 10.1175/JCLI-D-12-00430.1

[B21] Körner C. (2003). Carbon limitation in trees. J. Ecol. 91, 4–17 10.1046/j.1365-2745.2003.00742.x

[B22] Liu H. Williams A. P. Allen C. D. Guo D. Wu X. Anenkhonov O. A. (2013). Rapid warming accelerates tree growth decline in semi-arid forests of Inner Asia. Glob. Change Biol. 19, 2500–2510 10.1111/gcb.12217 23564688

[B23] Maness H. Kushner P. J. Fung I. (2013). Summertime climate response to mountain pine beetle disturbance in British Columbia. Nat. Geosci. 6, 65–70 10.1038/ngeo1642

[B26] McDowell N. G. (2011). Mechanisms linking drought, hydraulics, carbon metabolism, and vegetation mortality. Plant Physiol. 155, 1051–1059 10.1104/pp.110.170704 21239620 PMC3046567

[B25] McDowell N. G. Beerling D. J. Breshears D. D. Fisher R. A. Raffa K. F. Stitt M. (2011). The interdependence of mechanisms underlying climate-driven vegetation mortality. Trends Ecol. Evol. 26, 523–532 10.1016/j.tree.2011.06.003 21802765

[B27] McDowell N. G. Fisher R. Xu C. Domec J. C. Höltta T. Mackay D. S. (2013). Evaluating theories of drought-induced vegetation mortality using a multi-model-experiment framework. New Phytol. (in press). 10.1111/nph.12465 24004027

[B24] McDowell N. G. Pockman W. T. Allen C. D. Breshears D. D. Cobb N. Kolb T. (2008). Mechanisms of plant survival and mortality during drought: why do some plants survive while others succumb to drought? New Phytol. 178, 719–739 10.1111/j.1469-8137.2008.02436.x 18422905

[B28] Quirk J. McDowell N. G. Leake J. R. Hudson P. J. Beerling D. J. (2013). Increased susceptibility to drought-induced mortality in Sequoia sempervirens (Cupressaceae) under Cenozoic atmospheric carbon dioxide starvation, Am. J. Bot. 100, 582–591 10.3732/ajb.1200435 23425559

[B29] Raffa K. F. Aukema B. H. Bentz B. J. Carroll A. L. Hicke J. A. Turner M. G. (2008). Cross-scale drivers of natural disturbances prone to anthropogenic amplification: the dynamics of bark beetle eruptions. Bioscience 58, 501–517 10.1641/B580607

[B30] Sevanto S. McDowell N. G. Dickman T. L. Pangle R. Pockman W. T. (2013). How do trees die? A test of the hydraulic failure and carbon starvation hypotheses. Plant Cell Environ. [Epub ahead of print]. 10.1111/pce.12141 23730972 PMC4280888

[B31] Tardieu F. Simonneau T. (1998). Variability among species of stomatal control under fluctuating soil water status and evaporative demand: modelling isohydric and anisohydric behaviours. J. Exp. Bot. 49, 419–432

[B32] Thomas D. S. Eamus D. (1999). The influence of predawn leaf water potential on stomatal responses to atmospheric water content at consistent C_*i*_ and on stem hydraulic conductance and foliar ABA concentrations. J. Exp. Bot. 50, 243–251

[B33] Tyree M. T. Sperry J. S. (1989). Vulnerability of Xylem to Cavitation and Embolism. Ann. Rev. Plant Physiol. Plant Mol. Biol. 40, 19–36 10.1146/annurev.pp.40.060189.000315

[B34] van Mantgem P. J. Stephenson N. L. Byrne J. C. Daniels L. D. Franklin J. F. Fule P. Z. (2009). Widespread increase of tree mortality rates in the western United States. Science 323, 521–524 10.1126/science.1165000 19164752

[B36] Weiss J. L. Betancourt J. L. Overpeck J. T. (2012). Climatic limits on foliar growth during major droughts in the Southwestern USA. J. Geophys. Res. 117:G03031 10.1029/2012JG001993

[B35] Weiss J. L. Castro C. L. Overpeck J. T. (2009). Distinguishing pronounced droughts in the Southwestern United States: seasonality and effects of warmer temperatures. J. Clim. 22, 5918–5932

[B37] Will R. E. Wilson S. M. Zou C. B. Hennessey T. C. (2013). Increased vapor pressure deficit due to higher temperature leads to greater transpiration and faster mortality during drought for tree seedlings common to the forest-grassland ecotone. New Phytol. [Epub ahead of print]. 10.1111/nph.12321 23718199

[B38] Williams A. P. Allen C. D. Macalady A. K. Griffin D. Woodhouse C. A. Meko D. M. (2013). Temperature as a potent driver of regional forest drought stress and tree mortality. Nat. Clim. Change 3, 292–297 10.1038/nclimate1693

